# Particle Size Modulates Silver Nanoparticle Toxicity during Embryogenesis of Urchins *Arbacia lixula* and *Paracentrotus lividus*

**DOI:** 10.3390/ijms24010745

**Published:** 2023-01-01

**Authors:** Petra Burić, Ivana Čarapar, Dijana Pavičić-Hamer, Ines Kovačić, Lara Jurković, Maja Dutour Sikirić, Darija Domazet Jurašin, Nevenka Mikac, Niko Bačić, Daniel Mark Lyons

**Affiliations:** 1 Faculty of Natural Sciences, Juraj Dobrila University of Pula, Zagrebačka 30, 52100 Pula, Croatia; 2 Center for Marine Research, Ruđer Bošković Institute, Giordano Paliaga 5, 52210 Rovinj, Croatia; 3 Faculty of Educational Sciences, Juraj Dobrila University of Pula, Zagrebačka 30, 52100 Pula, Croatia; 4 Division of Physical Chemistry, Ruđer Bošković Institute, Bijenička cesta 54, 10000 Zagreb, Croatia; 5 Division of Marine and Environmental Research, Ruđer Bošković Institute, Bijenička cesta 54, 10000 Zagreb, Croatia

**Keywords:** dose-dependent, early life stage, embryonic development, exposure, kinetics, nanoparticle, silver ion

## Abstract

Silver nanoparticles represent a threat to biota and have been shown to cause harm through a number of mechanisms, using a wide range of bioassay endpoints. While nanoparticle concentration has been primarily considered, comparison of studies that have used differently sized nanoparticles indicate that nanoparticle diameter may be an important factor that impacts negative outcomes. In considering this, the aim of the present study was to determine if different sizes of silver nanoparticles (AgNPs; 10, 20, 40, 60 and 100 nm) give rise to similar effects during embryogenesis of Mediterranean sea urchins *Arbacia lixula* and *Paracentrotus lividus*, or if nanoparticle size is a parameter that can modulate embryotoxicity and spermiotoxicity in these species. Fertilised embryos were exposed to a range of AgNP concentrations (1–1000 µg L^−1^) and after 48 h larvae were scored. Embryos exposed to 1 and 10 µg L^−1^ AgNPs (for all tested sizes) showed no negative effect in both sea urchins. The smaller AgNPs (size 10 and 20 nm) caused a decrease in the percentage of normally developed *A. lixula* larvae at concentrations ≥50 µg L^−1^ (EC_50_: 49 and 75 μg L^−1^, respectively) and at ≥100 µg L^−1^ (EC_50_: 67 and 91 μg L^−1^, respectively) for *P. lividus*. AgNPs of 40 nm diameter was less harmful in both species ((EC_50_: 322 and 486 μg L^−1^, for *P. lividus* and *A. lixula*, respectively)). The largest AgNPs (60 and 100 nm) showed a dose-dependent response, with little effect at lower concentrations, while more than 50% of larvae were developmentally delayed at the highest tested concentrations of 500 and 1000 µg L^−1^ (EC_50_(100 nm); 662 and 529 μg L^−1^, for *P. lividus* and *A. lixula*, respectively. While AgNPs showed no effect on the fertilisation success of treated sperm, an increase in offspring developmental defects and arrested development was observed in *A. lixula* larvae for 10 nm AgNPs at concentrations ≥50 μg L^−1^, and for 20 and 40 nm AgNPs at concentrations >100 μg L^−1^. Overall, toxicity was mostly ascribed to more rapid oxidative dissolution of smaller nanoparticles, although, in cases, Ag^+^ ion concentrations alone could not explain high toxicity, indicating a nanoparticle-size effect.

## 1. Introduction

Over the past number of years, silver nanoparticles (AgNPs) have found a wide range of consumer and industrial applications, particularly due to their catalytic, optical and antibacterial properties [1]. Moreover, potential applications in biomedicine are increasing, following the development of strategies for tailoring their reactivity and stability through encapsulation in biocompatible coatings such as polyethylene glycol (PEG) which may aid cellular uptake [2,3]. However, it is their potential for release into the environment and negative impact on biota that is currently giving rise to concern. In terrestrial and aquatic environments, AgNPs can be stabilised by (dissolved) organic matter such as humic and fulvic acids [4]. Thus, AgNPs can remain relatively stable while passing through freshwater systems, such as lakes and rivers, and eventually reach marine environments [5,6]. While AgNPs have been classified as being extremely toxic (LC_50_ < 0.1 mg L^−1^) for relevant aquatic organisms [7], their modes of action are still being resolved. It is widely held that the release of Ag^+^ ions from the AgNP surface is the primary driver of toxicity [8], although there may be a non-negligible nanoparticle size effect. Several studies have demonstrated that AgNPs cause pro-oxidation and pro-inflammatory activity inside cells, either through released Ag^+^ ions or directly as a nanoparticle complex [9,10]. Research into AgNPs’ effect on environmentally relevant aquatic organisms has been focused mostly towards freshwater species, such as bacteria *Escherichia coli* and *Daphnia magna* [11]. However, there are increasing reports on their toxicity to marine organisms such as the Mediterranean mussel *Mytilus galloprovincialis* where they cause increased production of antioxidant enzymes [12], oyster embryos with increased metallothionein production [13] and also marine algae with inhibited growth [14,15]. Further, cnidaria *Aurelia aurita*’s larvae were affected by AgNPs such that exposure led to decreased larval movement in the water column [16].

Among models used for toxicity testing, the sea urchin has proven extremely valuable, particularly as it provides a wide range of endpoints for toxicity assessment and the functioning of its immune system is analogous to that of humans [17]. One of the most commonly used species in this bioassay is the purple sea urchin *Paracentrotus lividus*, mostly due to its very robust and observable morphological cleavages in the first stages of development [14,16,18,19]. Studies on the impact of nanoparticles on the urchin have found metal-oxide nanoparticles inside the immune cells of *P. lividus*, suggesting that the uptake into phagocytic coelomocytes might be a detoxification strategy used to promote organism survival [20]. Feeding the *P. lividus* larvae with metal-oxide nanoparticle-loaded microalgae was also found to alter rudiment growth, induce skeletal degradation and reduce larval viability [14]. Furthermore, cell death by apoptosis was noted in urchin embryos when exposed to amine-functionalised polystyrene nanoparticles, possibly due to cell membrane damage caused by the residual positive charge of this nanoparticle [21]. Moreover, copper- and zinc-oxide nanoparticles caused inhibition of ABC efflux transporters in the cell membrane, leading to their accumulation inside urchin embryos [22]. In addition, iron- and zinc-oxide nanoparticles were also found to pass the plasma membrane of ectodermal cells in *P. lividus* embryos, likely through endocytosis, with accumulation in cytoplasmic vesicles [23]. AgNPs have been shown to cause developmental defects in *P. lividus* embryos at specific concentrations [24], and we have previously shown that the effect of AgNPs on sea urchin embryonal development is not only dose-dependent, but is also species-specific and depends on the moment of first exposure. That study indicated that *P. lividus* embryos are the least sensitive species among the three species studied (including *Sphaerechinus granularis*, and *Arbacia lixula*) [19,25].

Considering that for a given mass of nanoparticles the total surface area increases inversely with size, and smaller nanoparticles have chemically more reactive surfaces due to a large surface-to-bulk atom ratio, it may be postulated that smaller nanoparticles may be more toxic than larger ones. In the case of AgNPs, this means a more rapid release of silver ions from smaller particles, leading to an increase in negative impacts on biota. While sea urchins occupy an important ecological niche, and are one of the most widely used models in embryology research due to their close analogies with human development, to the best of our knowledge there are no systematic studies quantifying the effects of such a wide range of nanoparticle sizes on both embryonic development and transmissible damage. Defining a size-based toxicological profile is all the more important considering increasing efforts to tailor nanoparticles’ size and surface chemistry for applications in biomedicine; for example, as antimicrobials and drug carriers. Therefore, the present study was designed to determine if AgNPs can negatively impact the early life stages of *A. lixula* and *P. lividus* in terms of toxicity to zygotes and to gametes (including transmissible damage to offspring), and quantify any correlation of toxicity with nanoparticle size. To give the broadest possible scope, five sizes of AgNPs (10, 20, 40, 60 and 100 nm) and concentrations ranging from 1 to 1000 µg L^−1^ were selected for examination.

## 2. Results

### 2.1. Nanoparticle Characterisation

**Figure 1:**
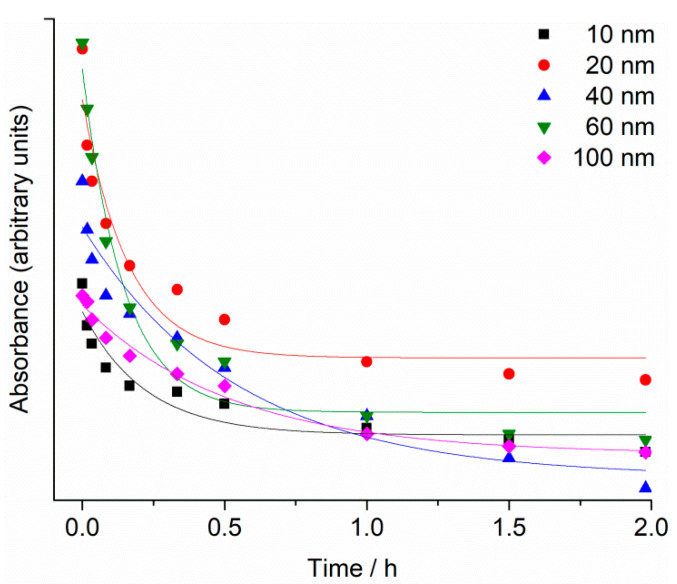
Temporal change in SPR maximum absorbance for 10, 20, 40, 60 and 100 nm AgNPs (1 mg L^−1^) in FSW.

UV-Vis absorption spectra of the as-purchased citrate-coated AgNP dispersions showed strong peaks, with maxima at 390 nm, 395 nm, 410 nm, 435 nm and 490 nm, which were assigned to the surface plasmon resonance (SPR) of the 10, 20, 40, 60 and 100 nm AgNPs, respectively (Supplementary Information; Figures S1–S5). The SPR absorption peak of AgNPs was observed to decrease over 48 h in filtered seawater (FSW) for all nanoparticle sizes, with the greatest reduction occurring within the first 20 min. (Figure 1). For example, SPR intensity for 40 and 60 nm AgNPs showed a decrease of about 50% within the first 20 min. after being dispersed in FSW, and is ascribed to initial rapid agglomeration of AgNPs in the high-strength electrolyte. In parallel, the SPR peaks were noted to gradually shift to lower wavelengths consistent with a reduction in particle size, likely due to oxidative dissolution.

The reduction of SPR intensity with time was modelled by an exponential decay function (*y = Ae^−x/t^ + y*^0^), with R^2^ value ranging from 0.91 to 0.98. Rate constants were similar for 10, 20 and 60 nm AgNPs, with values of 4.6 ± 1.6, 6.2 ± 1.9 and 6.9 ± 1.4 h^−1^, respectively, while for 40 and 100 nm AgNPs the rate constants were 1.8 ± 0.6 and 1.9 ± 0.3 h^−1^, respectively. 

**Figure 2:**
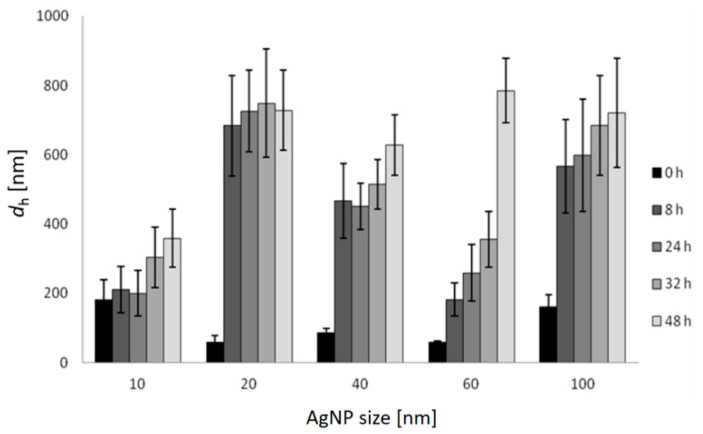
Hydrodynamic diameters (*d*_h_) and standard deviations of differently sized AgNPs in FSW over 48 h (*c* = 1 mg L^−1^) based on volume size distribution.

Changes in the hydrodynamic diameter (*d*_h_) of AgNPs over a period of 48 h in FSW are shown in Figure 2, and similar temporal trends were noted for all AgNPs sizes. The 10 nm AgNPs dispersion agglomerated immediately upon addition to salt water (FSW) and the first measurement at nominally t = 0 indicated the presence of a particle population of 180 nm diameter. During the following 48 h, these particles continued to show monomodal size distribution by volume in FSW, with the *d*_h_ increasing over time to 360 nm. AgNPs of 20 nm diameter initially showed a hydrodynamic diameter of 58 nm which increased to 683 nm, and remained relatively constant over time (up to 48 h). Similarly, agglomerates of 40 nm AgNPs increased in size from 87 nm to 631 nm over 48 h. More gradual increase in size of agglomerates was observed for 60 nm AgNPs, which increased in size from 59 nm to 181 nm over 8 h, and continued to 259 nm, 357 nm and 787 nm over 24, 32 and 48 h, respectively. The initial *d*_h_ of 100 nm AgNPs in FSW was 161 nm and quickly increased to 566 nm over 8 h, eventually attaining a size of 721 nm after 48 h. Thus, the hydrodynamic diameters of all agglomerates were significantly (ANOVA) larger than the diameters of the nanoparticles initially introduced to the FSW. While DLS was instrumentally limited to showing hydrodynamic diameters <6 μm, observation by light microscopy did not evidence nanoparticle aggregates larger than this over the course of the experiments.

**Figure 3:**
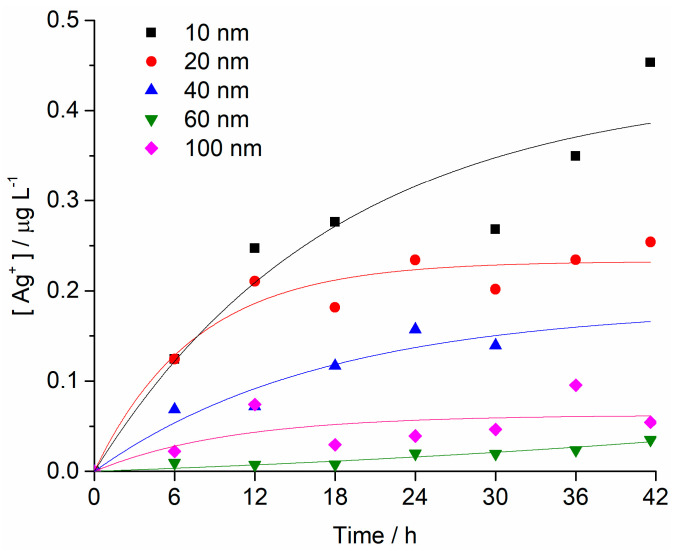
Concentrations of Ag^+^ ions as a function of time for all AgNP sizes in FSW (initial AgNP conc. 1 mg L^−1^). Each sample was measured twice, and RSD values were <5%.

The changes in concentration of Ag^+^ ions in FSW for different AgNPs sizes (starting concentration of 1 mg L^−1^) over time were determined by high-resolution inductively coupled plasma mass spectrometry. The fastest initial rate of increase in Ag^+^ ion concentration was noted for 10 nm AgNPs, which also showed the greatest ion concentration of 0.45 mg L^−1^ after 42 h. A similar rate of ion concentration increase was also observed for 20 nm AgNPs over the first 6 h, after which the rate of ion release slowed, with 25% of the total AgNPs mass eventually present as silver ions after 42 h. AgNPs with diameters of 40, 60 and 100 nm were noted to release Ag^+^ ions at progressively slower rates with increasing size. For example, after 42 h, the 60 nm AgNPs had released 4%, whereas 100 nm AgNPs released 5% of their mass as Ag^+^ ions (Figure 3).

### 2.2. Embryotoxicity

**Figure 4:**
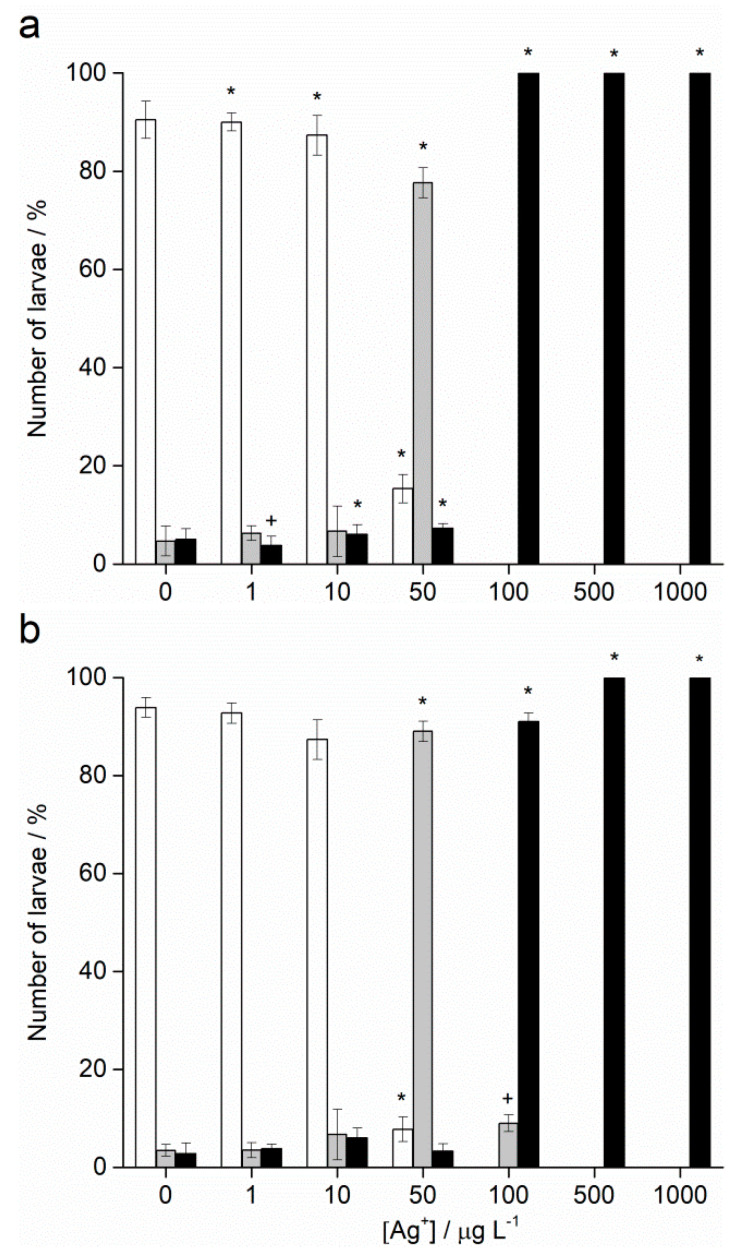
Percentage of normal (white bars) and developmentally delayed (grey bars) larvae, and undeveloped embryos (black bars) (±SD) of (**a**) *A. lixula* and (**b**) *P. lividus* exposed to various concentrations of Ag^+^ ions. Significance levels: ^+^ *p* < 0.05 and * *p* < 0.001.

Fertilisation success was >90% for non-treated control groups and was taken as indicating a valid test with appropriately healthy gametes. Zygotes of urchin species *A. lixula* and *P. lividus* exposed to a range of Ag^+^ ions showed similar responses in terms of normal, developmentally delayed or defective, and developmentally arrested embryos 48 h post-fertilisation (Figure 4). At concentrations of 1 and 10 µg L^−1^, Ag^+^ ions caused a statistically significant decrease of normally developed *A. lixula* larvae (90.0% and 87.3%, respectively) while *P. lividus* embryos treated with the same concentrations showed no significant differences in comparison with controls. A significant increase of larvae with developmental defects or developmentally delayed for *A. lixula* and *P. lividus* (77.7% and 89.0%, respectively) was noted after treatment with 50 µg L^−1^ Ag^+^ ions, with a concomitant reduction in normally developed larvae. Exposure to ionic silver concentrations in the range 100–1000 µg L^−1^ caused a block in embryonal development in both urchin species, with primarily dead zygotes observed.

**Figure 5:**
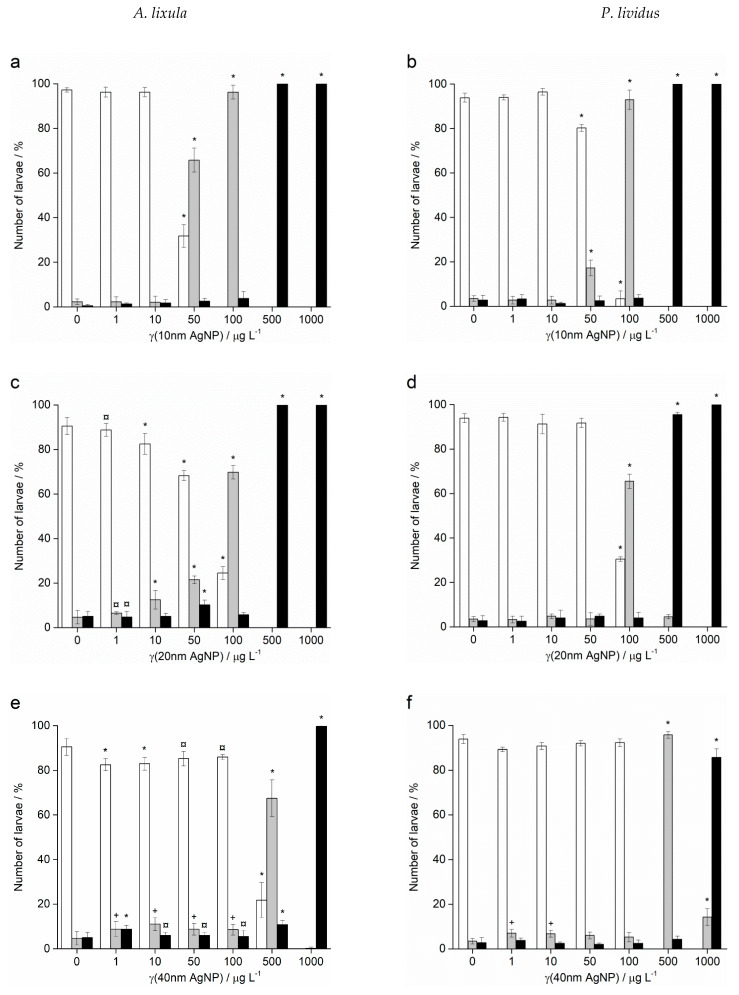

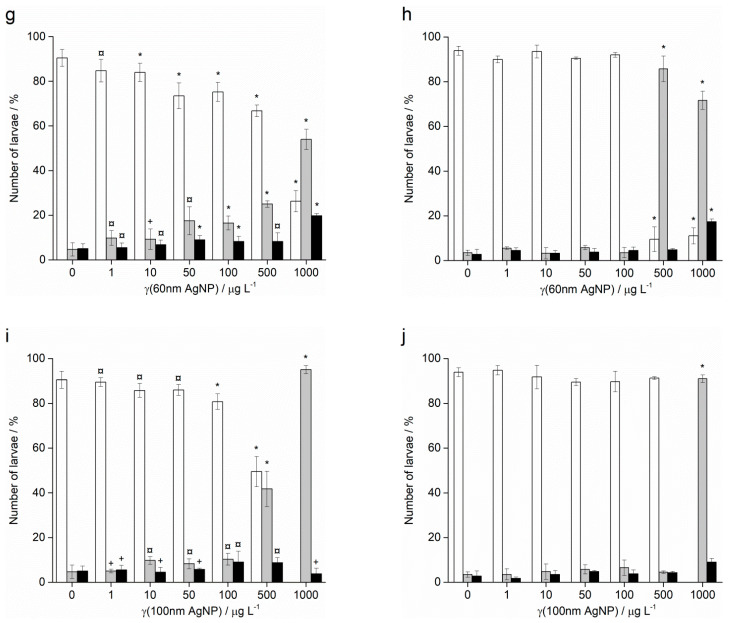
Percentage of normal (white bars) and developmentally delayed or defective (grey bars) larvae, and undeveloped embryos (black bars) (±SD) of urchins *A. lixula* (left bar charts) and *P. lividus* (right bar charts) exposed to AgNPs of diameter (**a**,**b**) 10 nm, (**c**,**d**) 20 nm, (**e**,**f**) 40 nm, (**g**,**h**) 60 nm, and (**i**,**j**) 100 nm. Significance levels: ^+^ *p* < 0.05, ^¤^ *p* < 0.01 and * *p* < 0.001.

In contrast to Ag^+^ ions, *A. lixula* embryos exposed to 1 and 10 µg L^−1^ of 10 nm AgNPs showed no difference in comparison to controls (Figure 5). However, the 10 nm AgNP concentrations of 50 and 100 µg L^−1^ gave a significant increase in defective larvae (65.8 and 96.3%, respectively), while concentrations of 500 and 1000 µg L^−1^ completely blocked normal development of the embryos (Figure 5a). Similarly, for 20 nm AgNPs, the percentage of normally developed *A. lixula* larvae decreased with increasing nanoparticle concentration (1–100 µg L^−1^), with a 4-fold decrease from 97.3% to 24.5% at 100 µg L^−1^ (Figure 5c). Again, the highest tested concentrations (500 and 1000 µg L^−1^) induced a complete block in embryo development. When exposed to 40 nm AgNPs *A. lixula* embryos showed a less pronounced effect, with ~10% decrease in the percentage of normal larva up to 100 µg L^−1^ concentration (Figure 5e). The 500 µg L^−1^ AgNP concentration induced a significant 4.5-fold decrease in the percentage of normally developed larvae, with an increase in defective and developmentally delayed larvae in parallel. Furthermore, 1000 µg L^−1^ of 40 nm AgNPs exposure resulted in only undeveloped embryos.

When the zygotes were treated with 60 nm AgNPs, a decrease in the percentage of normal larva was noted 48 h post-fertilisation with increasing silver concentrations from 1 to 1000 µg L^−1^ (Figure 5g). However, in comparison to the smaller-sized AgNPs (10, 20 and 40 nm), arrested development of embryos never exceeded 20%, while the majority of embryos were normally developed or showed some developmental defects. In particular, treatment of the zygotes at the highest concentration of 1000 µg L^−1^ resulted in mostly developmental delays and skeletal defects (54.0%), although not arrested development. In the 1–100 µg L^−1^ range, 100 nm AgNPs did not give rise to statistically significant differences from controls, while a concentration of 500 µg L^−1^ induced developmentally delayed larva (41.8%) that increased to 95.0% in zygotes treated with 1000 µg L^−1^ (Figure 5i).

As with *A. lixula* embryos, those of *P. lividus* exposed to silver concentrations of 1 and 10 µg L^−1^ for each tested AgNP size did not cause any significant growth anomalies in comparison to the non-treated larvae. The first decrease in normally developed larvae was noted when a concentration of 50 µg L^−1^ of 10 nm AgNPs was applied, with 80.3% normally developed larvae after 48 h (Figure 5b). The percentage of developmentally delayed larvae or those with skeletal defects significantly increased to 93.0% when the AgNP concentration was increased to 100 µg L^−1^. In contrast, AgNPs of 20 nm did not cause any effects significantly different from the non-treated controls, up to a concentration of 50 µg L^−1^, while an AgNPs concentration of 100 µg L^−1^ caused an increase of delayed or defective larvae to 65.5% (Figure 5d). Higher concentrations of both 10 and 20 nm AgNPs (500 and 1000 µg L^−1^) caused an almost complete block in embryo development (>96.0%).

As the nanoparticle size increased, it was noted that the number of normally developed larvae was not affected until increasingly high AgNP concentrations were applied. Specifically, for zygotes treated at 1–100 µg L^−1^ with 40 and 60 nm AgNPs, >90.0% of larvae were normally developed, while a concentration of 500 µg L^−1^ gave a significant increase in the number of developmentally delayed larvae (95.8 and 85.8%, respectively; Figure 5f–h). The highest concentration of 1000 µg L^−1^ of 40 nm AgNPs led to more than 80% arrested development while the 60 nm AgNPs resulted in most of the larvae showing developmental defects rather than arrested development (71.7%). The 100 nm AgNPs showed the least effect with nearly all larvae showing developmental delays rather than arrest at the highest concentration of 1000 µg L^−1^, while there were no significant differences from the controls for all other concentrations (Figure 5j). From the preceding embryotoxicity data EC_50_ and LC_50_ values were calculated for embryos of both urchin species, given in Table 1, using the sigmoidal dose–response function with variable Hill slope:

Y = A1 + (A2 − A1)/(1 + 10^(LOGx0-x)*p)^)

where A1 and A2 are the bottom and top asymptotes, respectively, LOGx0 is the centre, and p is the slope.

### 2.3. Spermiotoxicity

**Figure 6:**
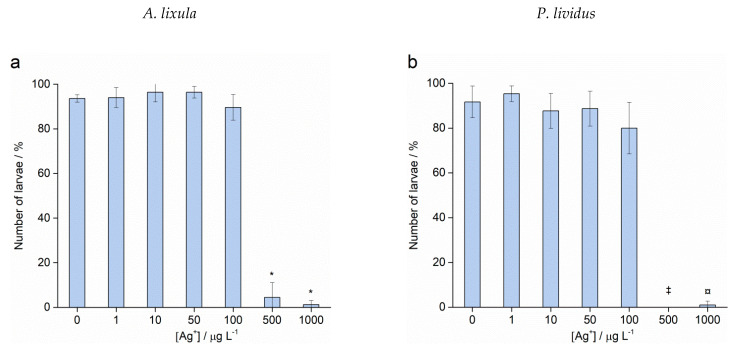
Fertilisation success for (**a**) *A. lixula* and (**b**) *P. lividus* after sperm pre-treatment with Ag^+^ ions (grey bars) and non-treated sperm (white bars) (±SD). Significance levels: ^¤^ *p* < 0.01, * *p* < 0.001, (‡ for the 500 µg L^−1^ treatment in *P. lividus*, no fertilised eggs were observed.

Fertilisation success was not affected after treatment of sperm with different sizes and concentrations of AgNPs for both species (data not shown). On the contrary, Ag^+^ ions caused a decrease in fertilisation success at the highest concentrations of 500 and 1000 μg L^−1^, resulting in only 4.4% and 1.2% zygotes undergoing cell division in *A. lixula* (Figure 6a), respectively, while nearly no fertilisation was observed for *P. lividus* at these Ag^+^ ion concentrations (Figure 6b).

**Figure 7:**
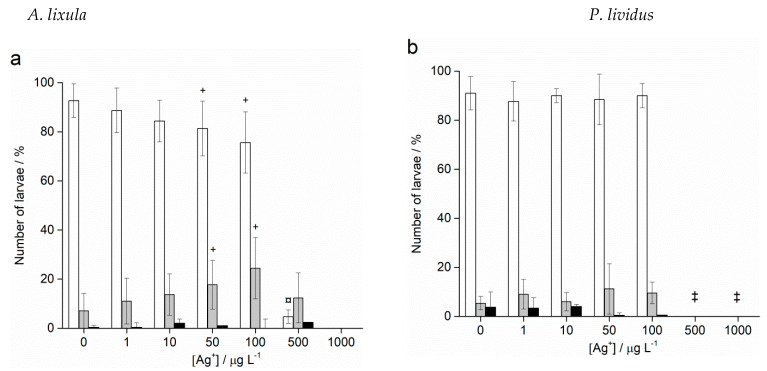
Offspring quality of (**a**) *A. lixula* and (**b**) *P. lividus* whose sperm was pre-treated for 1 h with Ag^+^ ions before fertilisation. White columns show normally developed larvae and gray columns developmentally delayed and defective larvae, while black columns indicate embryos with arrested development (±SD). Significance levels: ^+^ *p* < 0.05 and ^¤^ *p* < 0.01. (‡ only unfertilised eggs observed).

After sperm pre-treatment, those zygotes which were successfully undergoing cell division were analysed after 48 h for scoring of offspring quality. While no statistically significant differences to controls were observed for concentrations up to 10 μg L^−1^ of Ag^+^ ions, different sensitivity between the two urchin species was noted in the offspring of sperm pre-treated with 50 and 100 μg L^−1^ Ag^+^ ions, as shown in Figure 7. These concentrations resulted in a reduction of *A. lixula* offspring quality, which was expressed as a statistically significant increase (*p* < 0.05) in the number of developmentally delayed or defective larvae from 7.0% (in the control) to 17.9% and 24.5% in samples treated with 50 and 100 μg L^−1^ Ag^+^ ions, respectively. In contrast, the treatment of *P. lividus* sperm with the same Ag^+^ ion concentrations did not result in transmissible damage in the offspring compared to controls. The highest concentrations of Ag^+^ ions (500 and 1000 μg L^−1^) had already induced nearly complete failure of fertilisation in both species of urchin, hence there were no offspring to score except for a small number of *A. lixula* larvae (<100) whose sperm had been pre-treated with 500 μg L^−1^ Ag^+^ ion.

**Figure 8:**
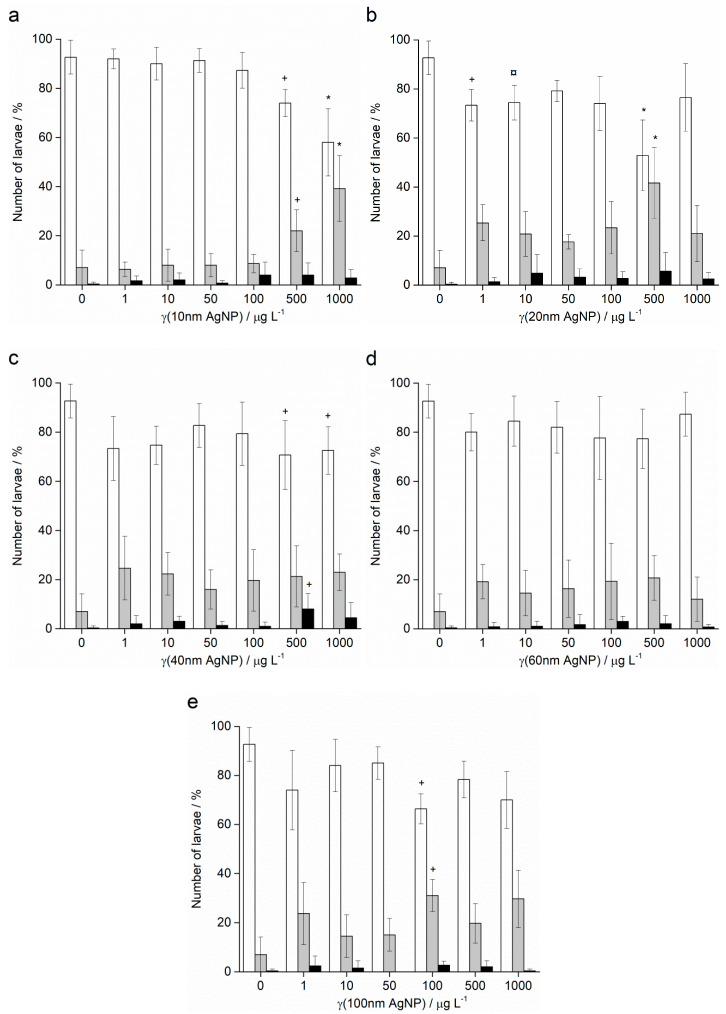
Offspring quality of urchins *A. lixula* whose sperm was pre-treated for 1 h with different sizes of AgNPs: (**a**) 10 nm, (**b**) 20 nm, (**c**) 40 nm, (**d**) 60 nm and (**e**) 100 nm. White columns indicate normally developed larvae and gray columns developmentally delayed or defective larvae, while black columns show embryos with arrested development (±SD). Significance levels: + *p* < 0.05, ¤ *p* < 0.01 and * *p* < 0.001 (‡ only unfertilised eggs observed).

Treatment of *A. lixula* sperm with 10 nm AgNPs at concentrations of 50 and 100 μg L^−1^ caused a decrease in the percentage of normally developed larvae to 81.5% and 75.3%, respectively, with a concomitant increase in larvae showing developmental defects or delayed development, while the number of embryos with arrested development remained very low and did not change significantly (Figure 8a). At the highest concentrations of 500 and 1000 μg L^−1^, few larvae were found, with a preponderance of developmental delays/defects and undeveloped eggs/zygotes, respectively. Similarly, the highest concentrations of 20 nm AgNPs also resulted in only undeveloped eggs being observed (Figure 8b). At concentrations in the range 1–100 μg L^−1^ pre-treatment of the sperm was not found to result in statistically significant differences from controls, with a high number of larvae displaying correct morphologies.

Pre-treatment of sperm with 40 nm AgNPs showed greater effects in offspring (Figure 8c). Specifically, the percentage of normally developed larvae after sperm pre-treatment with 1 μg L^−1^ AgNPs decreased from 91.9% in the control sample to 73.0%, and developmental defects increased from 7.2% to 25.5%. These levels remained relatively constant for all concentrations up to 100 μg L^−1^, while at the highest concentrations of 500 and 1000 μg L^−1^, the numbers of normal larvae observed were significantly lower at 69.8% and 72.1%, respectively. As the AgNP diameter increased to 60 nm (Figure 8d) and 100 nm (Figure 8e), no significant differences in offspring quality compared to controls was noted, except for an anomalous decrease in normal larvae (66.2%) and increase in developmental defects (31.1%) in the 100 μg L^−1^ treatment with 100 nm AgNPs.

**Figure 9:**
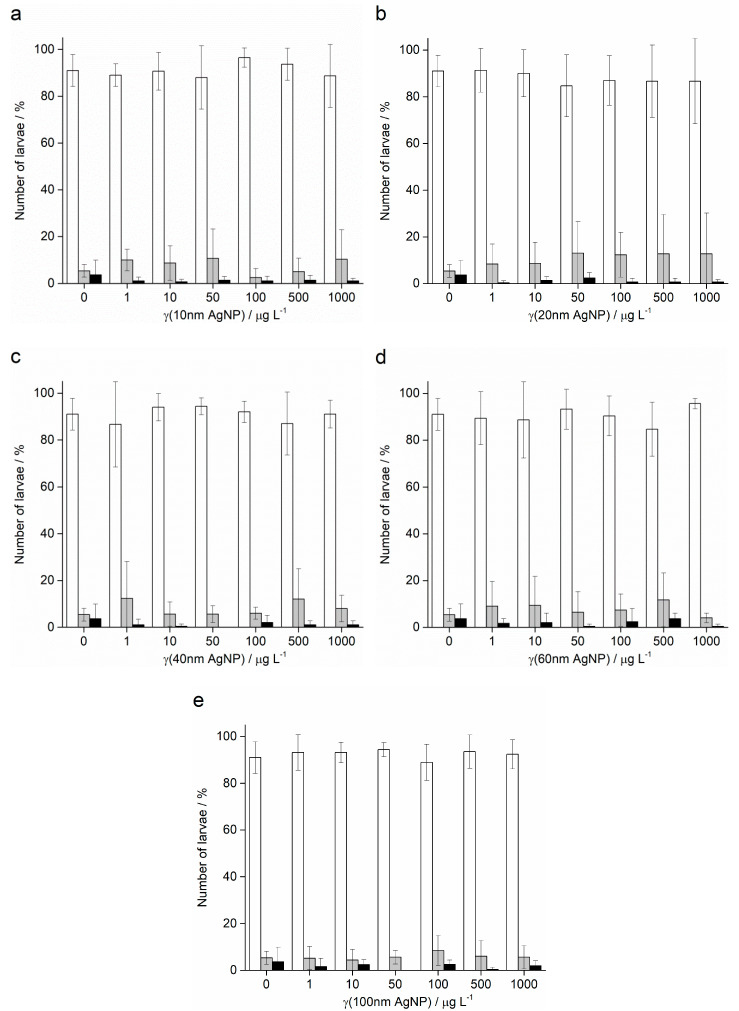
Offspring quality of urchin *P. lividus* whose sperm was pre-treated for 1 h with different sizes of AgNPs: (**a**) 10 nm, (**b**) 20 nm, (**c**) 40 nm, (**d**) 60 nm and (**e**) 100 nm. White columns indicate normally developed larvae and gray columns developmentally delayed or defective larvae, while black columns show embryos with arrested development (±SD).

In contrast to the effects of AgNPs on the offspring of pre-treated sperm of *A. lixula*, the pre-treatment of *P. lividus* sperm with AgNPs did not show a negative effect on the successful achievement of the pluteus larva stage. Namely, the offspring of sperm pre-treated with all sizes and concentrations of AgNPs successfully developed to the four-arm larval stage, in all cases achieving a high percentage of normally developed larvae (in the range 85–95%) that was not significantly different from the control (Figure 9).

## 3. Discussion

In this study, silver nanoparticles of varying diameter gave rise to a range of negative effects in urchins during embryogenesis, with clear concentration-dependent trends. In particular, the presence of smaller particles of 10 nm and 20 nm diameters led to an increase in negative outcomes, in terms of higher incidence of arrested development and developmental delays and defects, than larger particles of 60 nm and 100 nm at corresponding concentrations. Indeed, the smaller particles showed statistically significant effects at lower concentrations compared to the larger particles, while at higher concentrations a complete block in embryonic development was observed for these smaller particles. A similar concentration-dependent response to AgNPs was previously noted where treatment of zygotes with 0.6–30.0 × 10^13^ particles L^−1^ for 72 h caused a greater number of abnormally developed embryos of *P. lividus* with increasing concentration [26]. 

In a similar study on *P. lividus* it was found that citrate-coated 5–35 nm AgNPs induced developmental anomalies, such as asymmetry of the body (shorter arms) and changes in larval movement, at a concentration of 300 μg L^−1^ [24]. These results are similar to the ones obtained in this study for zygotes exposed to 500 μg L^−1^ of citrate-coated AgNPs with diameters of 10, 20 and 40 nm. Furthermore, the concentration of 50 μg L^−1^ of 10, 20 and 40 nm AgNPs herein did not cause any significant change in sea urchin embryonal development, consistent with previous results for 30 μg L^−1^ of 5–35 nm AgNPs [24]. After treatment of *P. lividus* embryos with AgNPs after 48 h, the developmentally delayed larvae were found to be swollen, and visible bright vesicles within the cytoplasm were also noticeable. Swelling, as well as other sub-lethal effects such as lethargy and immobilisation, were also reported in the echinopluteus and juvenile larval stages of urchin *Strongylocentrotus droebachiensis* exposed to 100 µg L^−1^ poly(allylamine)-coated silver nanoparticles (14 nm) for 24 h [27]. Ultimately, such swelling may be related to interaction of silver with the phospholipid double layer that can disrupt the transmembrane electrochemical gradient of the Na^+^ and K^+^ ions, eventually leading to disruption of the cell membrane potential and possible degradation of membrane integrity, leading to the observed “swelling” of the cell [9]. Similarly, carbon-based nanomaterials graphene oxide and carbon black have been shown to potentially disrupt the neurotransmitter acetylcholine functioning at gamete and zygote membranes in *P. lividus* [28], while CdS (7 nm) and ZnS (4 nm) semiconductor nanoparticles have also been shown to affect esterase activity and cause membrane depolarisation in different species of microalgae [29].

In addition to concentration dependence, the same sequence of experiments were carried out on *A. lixula* to determine if the response to AgNPs of different sizes is species dependent. In a previous study, this species was shown to be more sensitive to citrate-coated 60 nm AgNPs than urchins *P. lividus* and *Sphaerechinus granularis* over the concentration range 1–100 μg L^−1^ [19]. *A. lixula* embryos and larvae, after treatment with AgNPs, were reddish in colour, most likely because of an increase in the number of red coelomic spherulocytes, while in untreated larvae spherulocytes were uniformly distributed and much scarcer. In contrast, *P. lividus* red cells were also observed in treated and non-treated larvae; however, their quantity was much lower than in *A. lixula* larvae. It is possible that these spherulocytes are migrating to sites of inflammation caused by AgNPs. Infiltration of red spherulocytes to areas of injury in adult sea urchins was also observed, and it was suggested this might be part of the inflammatory response [30]. Indeed, nanoparticles including silver, titania, ceria, tin oxide, cobalt and iron oxide have all been shown to give rise to inflammation and generate oxidative stress in cells and tissues [31]. Swollen areas surrounded by a glossy membrane containing red granules and vibratile-like cells were also reported, and were attributed to a protective role in the preservation of organism homeostasis in adult sea urchin *S. purpuratus* [32]. Thus, it is reasonable to postulate that the same cellular defence mechanisms that occur as a response to AgNPs at early stages of development may also appear later in adult organisms. Indeed, the appearance of red spherulocytes in the pre-juvenile development of urchin larvae (up to 3 months of age) in response to 100 μg L^−1^ of 14 nm AgNPs was noted, whereas in post-larval stages the same were hardly observed [27]. However, as the development continued, the spherulocytes re-emerged in the post-metamorphic stages of the sea urchin development. 

AgNPs with decreasing diameters represent materials with increasing surface area per unit mass and greater surface activity by virtue of the increasing surface-to-bulk atomic ratio. In line with this, the interaction of smaller particles with biota may lead to more deleterious outcomes compared to larger nanoparticles, particularly due to biophysical interactions with membranes; for example, in various organisms and cells such as bacteria, yeast, algae and in vitro cancer cells [33]. In that study, it was found that 10 nm AgNPs more easily contacted with bacteria *E. coli* compared to larger particles, resulting in higher intracellular bioavailability of silver. Similarly, it was found that smaller polyvinylpyrrolidone (PVP) -coated AgNPs (10–40 nm) were more easily transported between (gill cell) epithelial layers of rainbow trout *Oncorhynchus mykiss* compared with larger AgNPs (100 nm) [34], while the greater toxicity of smaller AgNPs on mussel *M. galloprovincialis* haemocytes was also reported [35]. In a broader sense, size-dependent toxicity for nanoparticles is not limited to AgNPs as a range of other nanoparticles have been found to show similar effects, where smaller sizes result in more negative outcomes in bioassays. For example, it was noted that the effect of silica nanoparticles (SiNP) on the marine alga *Chlorella kessleri* was dependent on the nanoparticle size [36]. Specifically, SiNP of diameters 5 nm and 26 nm showed greater toxicity in the microalga than those with larger diameters (78 nm), while similar differentiation was noted in human kidney cells for particles of diameter 20 nm and 100 nm [37].

The lower toxicity of larger nanoparticles, and AgNPs in particular, may in part be related to the fact that they can be removed from the cell by mechanisms of facilitated transport of larger cell aggregates, such as by multi-xenobiotic resistance proteins (MXR-proteins) and ABC transporter proteins [38]. Such outcomes are in line with other reports that found retention of smaller diameter 50 nm AgNPs for a longer period of time in the body of endemic flea *Glyptotendipes tokunagai*, while larger particles (100 and 150 nm) were taken up and excreted from the body relatively quickly [39]. Similarly, it was demonstrated by X-ray microanalysis that 5 nm gold nanoparticles (AuNP) accumulated in greater amounts in the digestive gland of mussel *M. galloprovincialis* compared with 40 nm AuNP [40]. Furthermore, the high salt concentration in estuarine and coastal waters is an important factor that impacts on the behaviour of AgNPs, with these high strength electrolytes causing disruption of the electric double layer around the nanoparticles, thus facilitating agglomeration. In their interaction with organisms, it is possible that larger aggregates are easier to take up from the water column than individual nanoparticles; for example, as shown in the accumulation of AgNPs in mussel gills [41]. Moreover, the formation of aggregates does not necessarily lead to reduced bioavailability of nanoparticles; AgNPs from wastewater remain bioavailable, regardless of the aggregates formed, and consequently cause a range of inflammatory processes in fish [42]. In the present work, at the very high concentrations of 500 and 1000 μg L^−1^, there are fewer normal larvae after exposure to 100 nm compared with 60 nm, which is the opposite of what we would expect and not in line with the very consistent results for all the other nanoparticle sizes and concentrations. It may be that, at such high concentrations, the larger 100 nm particles, with or without agglomeration, are more easily taken up from the exposure medium, particularly in the latter stages of larval development when the gut is fully formed and the plutei actively begin to feed. However, assigning such a reason to the observation of fewer normally developed plutei after exposure to high concentrations of 100 nm silver nanoparticles is admittedly speculative, and more detailed investigation is required before a definitive answer may be offered. Dynamic light scattering data indicated that the AgNPs rapidly agglomerated when placed in filtered seawater, due to the high salt concentrations; although, small populations of individual nanoparticles persisted in parallel with the large agglomerates. UV-Vis absorption data also showed rapid agglomeration of the AgNPs; yet the SPR, though low intensity, remained visible until the end of the experiment, also suggesting that individual particles or very small agglomerates were still present and likely stabilised by dissolved organic matter [4,43]. Consequently, it is likely that urchin gametes or zygotes were exposed to both individual nanoparticles and large agglomerates over 48 h of the experiment.

Apart from availability of such particles and agglomerates for uptake, exposure to silver ions represents a third factor that impacted upon AgNP toxicity. Specifically, smaller AgNPs release ions from their surfaces more rapidly compared to those of larger dimensions, which can play an important role in determining overall toxicity in the tested organism [44]. While released silver ions are widely considered to be the primary driver of toxicity of AgNPs, there are cases where AgNPs have shown themselves to be more toxic than the corresponding mass of silver in ionic form [19]. This is all the more striking, considering that equal masses of silver in ionic and nanoparticulate form in solution do not have the same quantity of free ionic silver, but rather, the nanoparticle has significantly less since it releases silver ions slowly. As noted previously, after 42 h, the silver ion concentration measured in a 1 mg L^−1^ dispersion of 100 nm AgNPs was 0.05 mg L^−1^, indicating 5% dissolution of the nanoparticles. Thus, for example, it may be more prescient to compare the toxicity of the 1000 μg L^−1^ AgNPs (100 nm) treatment with the 50 μg L^−1^ Ag^+^ treatment. Comparing the effects of these two treatments on embryotoxicity, the nanoparticles resulted in 95% delayed-development or defective larvae, while the Ag^+^ treatment gave rise to 78% delayed or defective larvae. In the former, no normally developed larvae were observed, while for the Ag^+^ treatment, 15% of the larvae were normal. This seems to indicate that silver ions are not the only mechanism for silver toxicity, but that there is a nanoparticle effect, possibly related to biophysical interactions with membranes and nanoparticle internalisation which may lead to increased silver ion availability in the organism. Similarly, the greater toxicity of 10 nm AgNPs on bacteria *E. coli* was attributed to the bioavailability and easier internalisation of NP compared to Ag^+^ ions [33]. Moreover, in the present work, the first significant differences from controls in the *P. lividus* embryotoxicity test for both the 10 nm AgNPs and Ag^+^ treatments was noted at mass concentrations of 50 μg L^−1^. However, the 10 nm AgNPs showed only 45% dissolution by the end of the experiment, indicating that the nanoparticle/ion combination may be more toxic to developing embryos than just ionic silver alone. Similar results showing a stronger negative effect on sea urchin *P. lividus* embryonic development was due to AgNPs [24]. Negative effects of AgNPs on the embryonic development of oyster *Crassostrea virginica* have been reported, although toxicity was not attributed solely to AgNPs but also to the Ag^+^ ions present [13]. Furthermore, AgNPs coated with glucose showed a similarly higher embryotoxicity toward sea urchin embryos compared to Ag^+^ ions [26]. Contrary to the aforementioned studies, higher toxicity of Ag^+^ ions versus 15 nm PAAm-AgNPs were noted during early-stage development of the urchin *Strongylocentrotus droebachiensis*, which attributed developmental defects and lethargy rather to ion exposure than nanoparticles [45]. However, exposure to nanoparticulate and ionic silver gave rise to distinct effects, as the silver uptake mechanism depended on both forms of silver being present and the life stage in *S. droebachiensis*. In particular, it was noted that exposure to 15 nm PAAm-AgNPs gave rise to a range of effects, including oedema, necrosis [46], induction of heat-shock protein expression and generation of oxidative stress [27].

As an additional factor that must be considered in nanoparticle toxicity is the shape of the nanoparticles in contact with biota, which has been shown to be important. For example, the effect of various AgNPs forms on the RT-W1 California trout cell line (*O. mykiss*) was investigated and it was found that nanoplates are the most toxic form of AgNPs compared with spheres and tubes [47]. In that study, a large number of crystal anomalies (stacking faults and point defects) were observed by electron microscopy on the silver nanoplate surface which represent locations where surface silver atoms may be more easily released through oxidative dissolution.

Considering that not only developing zygotes and pluteus larvae may come in contact with AgNPs but also gametes, a series of experiments to probe the effect of the different sizes of AgNPs on sperm’s ability to successfully fertilise eggs was carried out. The results showed that, irrespective of size, AgNPs did not affect the fertilisation success in *A. lixula* and *P. lividus* after pre-treatment of sperm with AgNPs for one hour. A similar result was noted after *P. lividus* sperm exposure to 1–10 nm AgNPs [16]. Furthermore, it was found that the addition of AgNPs to the medium containing sea urchin gametes does not interfere with rising of the fertilisation membrane [26]. However, asymmetric cell division in the embryo and, to a lesser extent, the absence of the division was subsequently observed. Contrary to these results, carbon NP (graphene oxide and black carbon), due to the physical interaction between carbon nanomaterials and sperm, significantly reduced the fertilisation success of treated *P. lividus* sperm [28]. In the present study it was determined that silver ion concentrations up to at least 100 μg L^−1^ did not negatively impact fertilisation success.

The quality of the offspring of the pre-treated sperm was scored after 48 h and for the 10 nm AgNPs there was a reduction in the offspring quality at concentrations of (≥50 μg L^−1^), while for larger AgNP sizes this concentration threshold increased. It was previously reported that 40 nm AgNPs were internalised in mouse sperm, followed by reduced fertilisation ability and a lower percentage of normally developed embryos [48]. So, while we might expect a greater degree of internalisation of smaller 10 nm and 20 nm AgNPs with less fertilisation success and a greater degree of offspring damage compared with 40 nm and larger particles, such an effect was generally not observed for lower AgNP concentrations. It may be that for low concentrations, mechanisms for small particle removal were dominant; for example, by exocytosis or via efflux protein transfer. At the higher concentrations of 10 nm, 20 nm and 40 nm AgNPs (500–1000 μg L^−1^) a concentration-dependent negative effect was noted on larval development, which may be a consequence of more ready uptake of larger particles and agglomerates through an efflux mechanism as discussed previously. As with *A. lixula*, treatment of *P. lividus* sperm with various sizes of AgNPs did not lead to a negative effect on fertilisation success and the offspring quality was not significantly different from controls. In contrast, it was observed that 1–10 nm AgNPs have a negative effect on the larval development of sea urchin *P. lividus* after sperm exposure to the AgNPs in the concentration range 0.1–1000 μg L^−1^ [16].

Overall, it is clear from the data presented herein that *A. lixula* zygotes and larvae are more susceptible to the presence of silver, in both ionic and nanoparticulate form, than *P. lividus*. This result is closely in line with a previous study which highlighted the fact that more sensitive species should be selected in ecotoxicity testing, as more robust species may give false negatives in terms of the toxicity of specific materials [19]. Why there is such a clear difference in effect between the two species may be a matter of speculation. For example, should *A. lixula* need to build a much harder and more complex skeleton than *P. lividus*, then differences in the biomineralisation process can point to why AgNPs have a different toxicity profile in *A. lixula* compared with *P. lividus* [49]. In that study, it was reported that sensitivity to gadolinium (Gd) could reflect differences in the calcium concentration in the more complex skeleton compared with a simpler skeleton presenting a lower calcium concentration (such as in *P. lividus*). Therefore, it is possible that urchins that have a more prodigious uptake of calcium can be affected to a greater degree by some pollutants; for example, Gd and AgNPs, ultimately interfering with Ca^2+^ uptake. This again highlights the need to carefully select the model used in bioassays, considering how their susceptibility to different environmental pollutants may vary. In broader terms, insights from these data on the physicochemical and toxicological size-dependent behaviour of silver nanoparticles may also be of use in areas such as biomedicine and mariculture, for example in cancer treatment [50] and mariculture fish feeds [51], where stability and dissolution kinetics are important factors that impact upon the therapeutic profile of the nanoparticles. Furthermore, by implementing strategies for controlling nanoparticle morphology, their physico-chemical behaviour in media such as physiological fluids or marine waters may be modulated to provide tailored solutions in these fields of application [52].

## 4. Materials & Methods

### 4.1. Materials

Various sizes of spherical AgNPs (diameters: 10, 20, 40, 60 and 100 nm; conc. 20 mg L^−1^) and silver nitrate at the highest analytical grade were purchased from Sigma-Aldrich (St. Louis, MO, USA), while potassium chloride and potassium chromium (III) sulphate (p.a) were purchased from Kemika (Zagreb, Croatia). Silver and rhodium ICP-MS standards, at a concentration of 1000 mg L^−1^ in 2% nitric acid, were purchased from Absolute Standards Inc. (Hamden, CT, USA). The nitric acid used was 67–69% Ultrapure for trace analysis (CARLO ERBA Reagents S.r.l., Milan, Italy). Filtered seawater (FSW) was obtained by filtering natural northern Adriatic Sea water (salinity S·38.1 ± 0.1, pH 8.1 ± 0.1) through 0.22 µm pore polycarbonate-membrane filters (Whatman Cyclopore; Little Chalfont, UK) while ultrapure water (18 MΩ·cm) was obtained from a TKA GenPur system (TKA Wasseraufbereitungssysteme GmbH, Niederelbert, Germany). 

### 4.2. Nanoparticle Characterisation

Ultraviolet-visible absorption spectroscopy (UV-Vis) was used to track evolution of the surface plasmon resonance of AgNP dispersions (initial concentration of 1 mg L^−1^) in FSW over time. Absorption spectra of AgNPs were recorded on a Shimadzu UV1800 dual-beam spectrophotometer in the wavelength range 200–800 nm, with a resolution of 1 nm, in quartz cuvettes with a 10 mm optical path. Data were processed using UVProbe 2.3.1 (Shimadzu, Kyoto, Japan) and Origin 9.0 (OriginLab Corporation, Northampton, MA, USA) software. Dynamic light scattering (DLS) was used to determine particle size and agglomeration processes in FSW over 48 h. Data were collected on a Zetasizer Nano ZS instrument (Malvern Instruments, UK) equipped with a green laser (532 nm), with samples held in 10 mm polystyrol/polystyrene cuvettes, and intensity of scattered light was detected at an angle of 173º. Samples were measured 10 times and the data were processed on Zetasizer 6.32 software (Malvern Instruments, UK). The results are reported as hydrodynamic diameters (*d*_h_) based on the calculated volume size distributions. To determine total amount of Ag^+^ ions in FSW released from AgNPs over time, a series of AgNP dispersions in FSW (1 mg L^−1^) were prepared and filtered after different time periods (6, 12, 18, 24, 30, 36, 42 and 48 h) in Vivaspin 3 kDa MWCO centrifugal filters (Merck KGaA, Darmstadt, Germany). Silver concentrations in the filtrates were determined by high-resolution inductively coupled plasma mass spectrometry on an Element 2 (Thermo, Germany) instrument with SC-2 DX FAST autosampler (Element Scientific, USA) as the sample introduction system. After ultrafiltration, the filtrates were diluted 20 times with dilute nitric acid (2% *v*/*v* TraceSELECT; Fluka, Buchs, Switzerland) and indium (Fluka) was added as the internal standard (1 μg L^−1^). Two isotopes of silver (^107^Ag and ^109^Ag) were measured. Standard solutions were prepared by appropriate dilution of a stock solution of silver (Fluka, 1.000 g L^−1^) in 2% v/v nitric acid. Ag^+^ ion concentrations in all filtrates were measured in duplicate (<5% RSD).

### 4.3. Embryotoxicity

**Figure 10:**
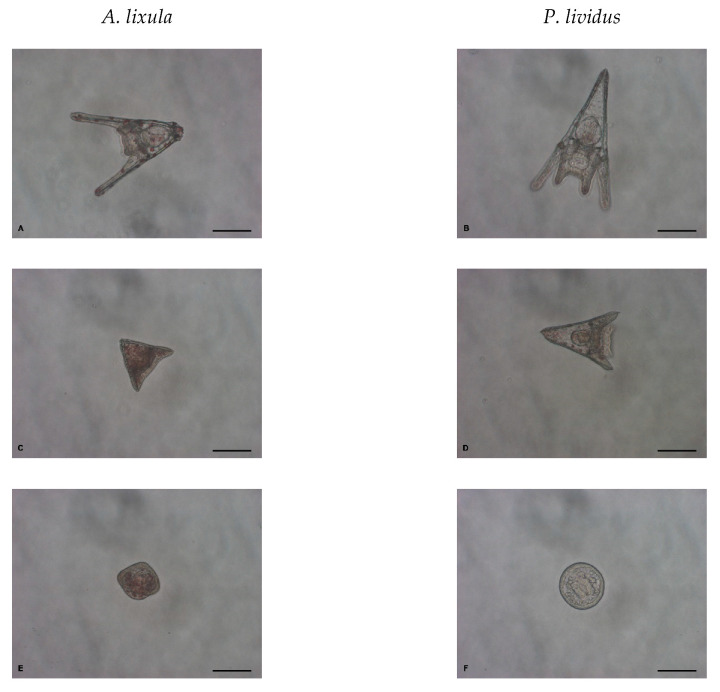
Normal larvae at pluteus stage (**A**,**B**), developmentally delayed larvae (**C**,**D**) and undeveloped embryos (**E**,**F**) after 48 h exposure to AgNPs (scale bar = 100 μm).

Adult Mediterranean sea urchins *A. lixula* and *P. lividus* were collected along the northern Adriatic coast near Pula, Croatia (44°51′20″ N, 13°49′33″ E) and transported within 1 h to outdoor aquaria with running natural sea water in a flow-through system. The sea urchins were used within five days of collection and the experiments were typically carried out in April. The sea urchin embryo-development test was performed as previously described with small modifications [19]. Briefly, gametes were collected by injecting 1 mL of 0.5 M KCl aqueous solution through the peristomal membrane of 2 male and 3 female sea urchins. Fertilisation was carried out in 500 mL FSW by adding egg stock suspension, such that the final concentration was ~100 cells mL^−1^, and 0.25 mL diluted sperm (×10 in FSW). The sperm of each male was used to fertilise the eggs of each of the three females to give 6 replicates (Supplementary Information: Figure S6). Approximately 2 h post-fertilisation, at the 4–8 cell stage, the embryos were transferred to polystyrene six-well tissue culture plates (10 mL well^−1^) and treated with 10, 20, 40, 60 and 100 nm AgNPs or Ag^+^ ions (AgNO_3_ solution). The final concentrations of AgNPs and Ag^+^ ions (as mass of silver) were 0, 1, 10, 50, 100, 500 and 1000 μg L^−1^. Developing embryos were kept at 20 °C and the plates were held in the dark to avoid any possible influence of sunlight on the AgNPs and Ag^+^. After 48 h, the larvae were immobilised with 10^−4^ M chromium (III) sulphate 10 min. prior to observation [53] and observed for developmental abnormalities using a Carl Zeiss Axiovert 200 inverted microscope equipped with an AxioCam MRc5 camera. The first 100 larvae in each of the six replicates were scored for the percentage of: (1) normal larvae at pluteus stage with two fully developed arms and legs, and round translucent gut, (2) developmentally delayed larvae at least two times smaller than the control or with some other anomalies not seen in the non-treated control (i.e., shorter arms or legs, fused arms, crossed or separated tips, black gut), and (3) undeveloped embryos (i.e., arrested development at the morula, blastula or gastrula phase) (Figure 10). As test validation, control samples of non-treated embryos for both species showed more than 95% normally developed larvae after 48 h.

### 4.4. Spermiotoxocity

The spermiotoxicity assay was carried out as previously described [54]. Sperm, collected ‘dry’ by Pasteur pipette upon release from the urchin test, was diluted ×10^3^ in FSW, and AgNPs of different sizes, or Ag^+^ (as AgNO_3_), were added such that the final Ag concentrations were in the range 1–1000 μg L^−1^. Sperm was thus exposed to AgNPs or Ag^+^ ions for 1 h. Subsequently, 100 μL aliquots of pre-exposed sperm (from 2 males) were used to fertilise eggs from 3 females (~100 cells mL^−1^) in the wells of six-well tissue culture plates (10 mL well^−1^) to give 6 replicates (Supplementary Information: Figure S7). Fertilisation success was determined 2 h post-fertilisation as the percentage of embryos undergoing cell division. After two days, the quality of the offspring was quantified by scoring larval development, as previously described, for the embryotoxicity assay.

### 4.5. Statistical Analysis

Statistical analysis was carried out by analysis of variance (ANOVA), followed by the Bonferroni post hoc test, after testing the data for normality (Shapiro-Wilk test) and homogeneity of variance (Levene test). The significance levels are denoted as ^+^ *p* < 0.05, ^¤^ *p* < 0.01 and * *p* < 0.001. Data processing was carried out on Origin v9.0 (OriginLab Corporation, Northampton, MA, USA) software.

## 5. Conclusions

Early-life stages of sea urchins have proven to be useful models for uncovering the effects of AgNPs and how size modulates nanoparticle toxicity. Regardless of their size, AgNPs tend to quickly agglomerate in seawater and ion release by oxidative dissolution proceeds more rapidly than in freshwater. Thus, the system presents a combination of ionic and particulate silver to developing embryos, which likely impacts on the organism by different mechanisms, such as endo/exo-cytosis or via efflux protein transfer. It was confirmed that the kinetics of ion release depends on the particle size, as for a given mass of AgNPs the smaller particles have a greater total surface area and the ratio of surface–to–bulk atoms is high, resulting in more ready release of ions from the surface. This more rapid ion release correlates with an increase in toxic effects in urchin embryos that manifest as developmental defects, delayed development and arrested development. However, silver ions could not explain the negative outcomes during urchin development in every case, and it is likely that a nanoparticle-size effect was responsible; for example, through disruption of membranes and enhanced uptake followed by subsequent silver ion delivery/release within cells. Interestingly, while AgNPs were not found to impact fertilisation, transmissible damage to offspring was noted in *A. lixula*. Overall, it may be concluded that sea urchin *A. lixula* is more sensitive to the presence of ionic and particulate silver compared with *P. lividus* species and may be a preferable model for nanoparticle toxicity testing than the commonly used *P. lividus*.

## Figures and Tables

**Table 1:** Calculated EC_50_ and LC_50_ values (±SE) for *A. lixula* and *P. lividus* plutei exposed to AgNPs of different sizes for 48 h.

	Diameter/nm	EC_50_/μg L^−1^	R^2^	LC_50_/μg L^−1^	R^2^
*A. lixula*	10	48.59	0.99	387.34 ± 10.13	0.98
	20	74.90 ± 2.90	0.99	397.67 ± 2.00	0.99
	40	485.57	0.99	903.08 ± 17.61	0.99
	60	846.77 ± 67.63	0.98		
	100	529.11 ± 21.54	0.99	1433.97	0.99
	Ag^+^	39.50 ± 2.33	0.99	87.95	0.99
*P. lividus*	10	66.87 ± 0.95	0.99	327.25	0.99
	20	91.25 ± 10.43	0.97	376.34 ± 10.00	0.99
	40	322.45	0.99	724.06 ± 25.48	0.98
	60	360.88 ± 189.72	0.99		
	100	662.26	0.99		
	Ag^+^	29.29 ± 0.80	0.99	73.79 ± 1.26	0.99

## Data Availability

Not applicable.

## Supplementary Materials

The following supporting information can be downloaded at: https://www.mdpi.com/article/10.3390/ijms24010745/s1.
