# Supplementary material for: Particle Size Modulates Silver Nanoparticle Toxicity during Embryogenesis of Urchins Arbacia lixula and Paracentrotus lividus
Source: Int J Mol Sci. 2023 Jan 1;24(1):745. doi: 10.3390/ijms24010745 (PMC9821580; doi:10.3390/ijms24010745)
Supplement: Supplementary file 1 [file ijms-24-00745-s001.zip › ijms-2105039-supplementary.pdf]

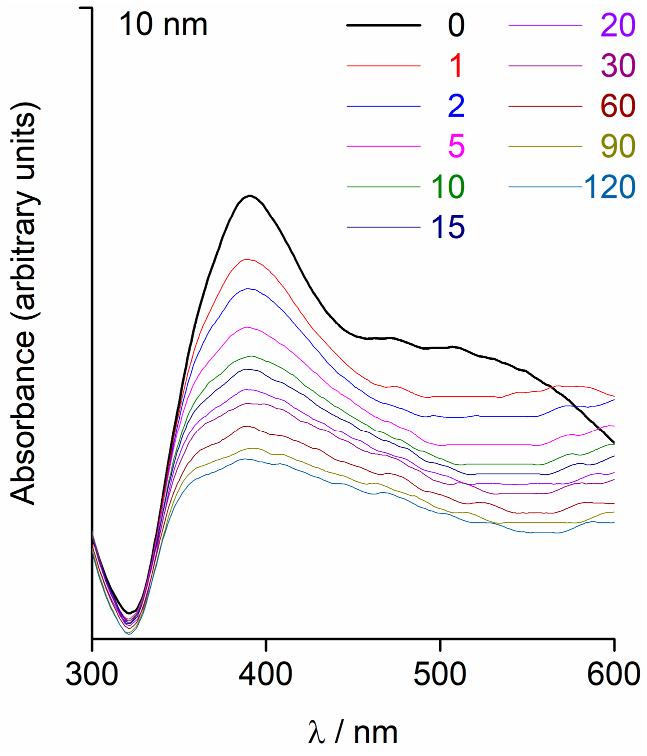

Figure S1. Temporal change in surface plasmon resonance for 10 nm AgNPs (1 mg L^{-1}) in FSW. Legend shows time in minutes after addition of AgNP to FSW.

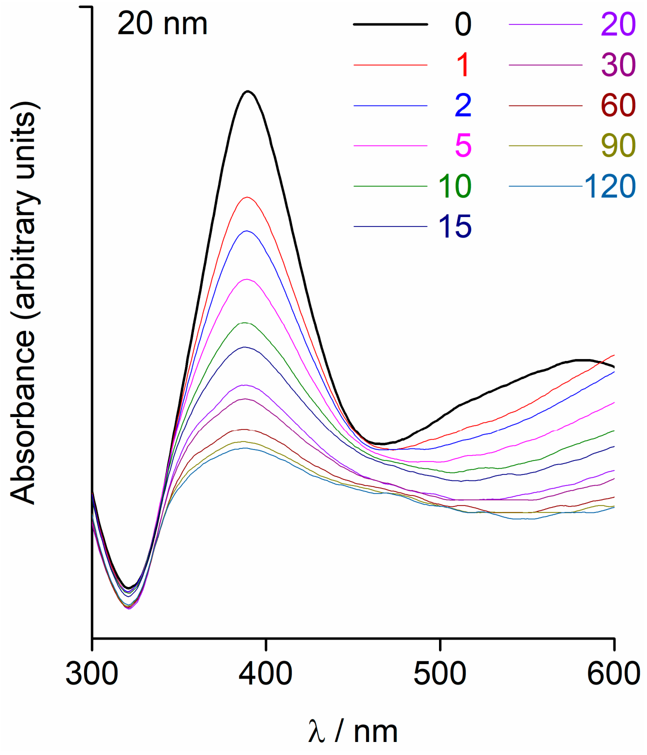

Figure S2. Temporal change in surface plasmon resonance for 20 nm AgNPs (1 mg L^{-1}) in FSW. Legend shows time in minutes after addition of AgNP to FSW.

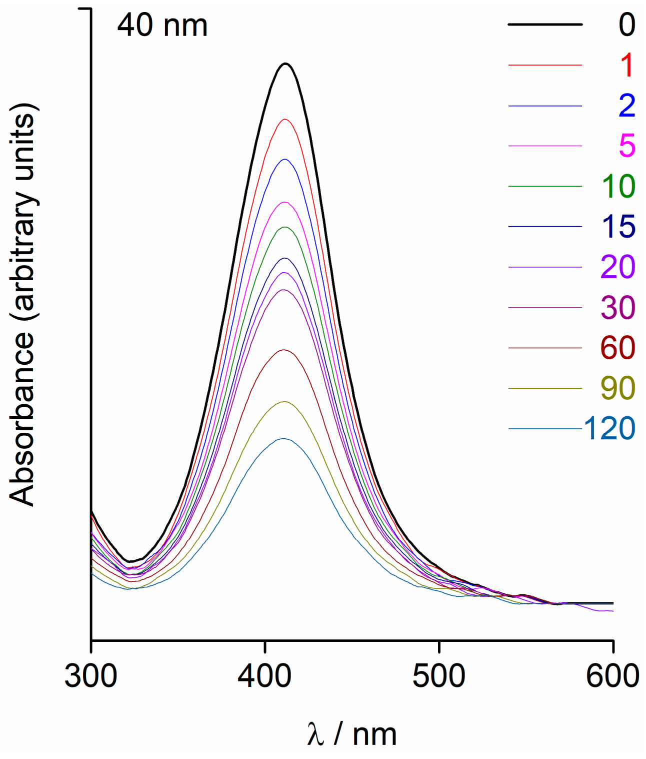

Figure S3. Temporal change in surface plasmon resonance for 40 nm AgNPs (1 mg L^{-1}) in FSW. Legend shows time in minutes after addition of AgNP to FSW.

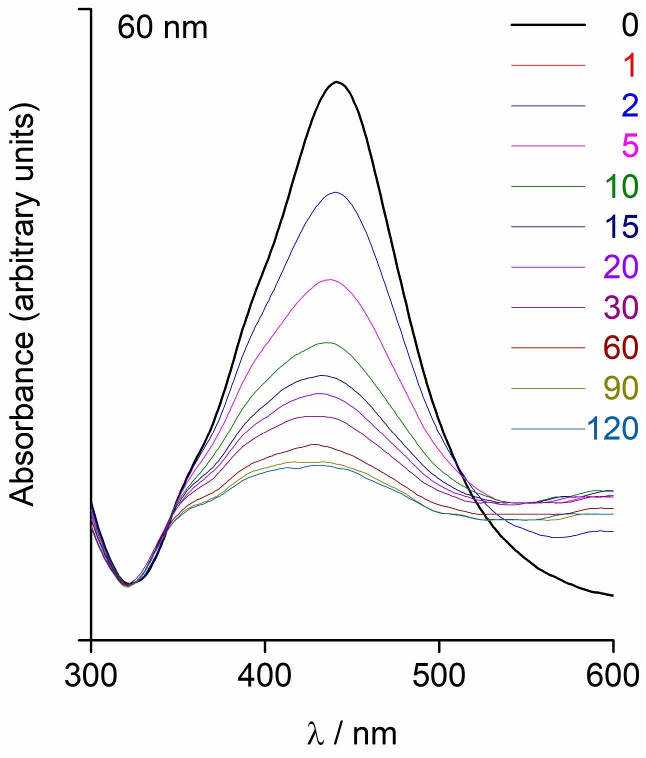

Figure S4. Temporal change in surface plasmon resonance for 60 nm AgNPs (1 mg L^{-1}) in FSW. Legend shows time in minutes after addition of AgNP to FSW.

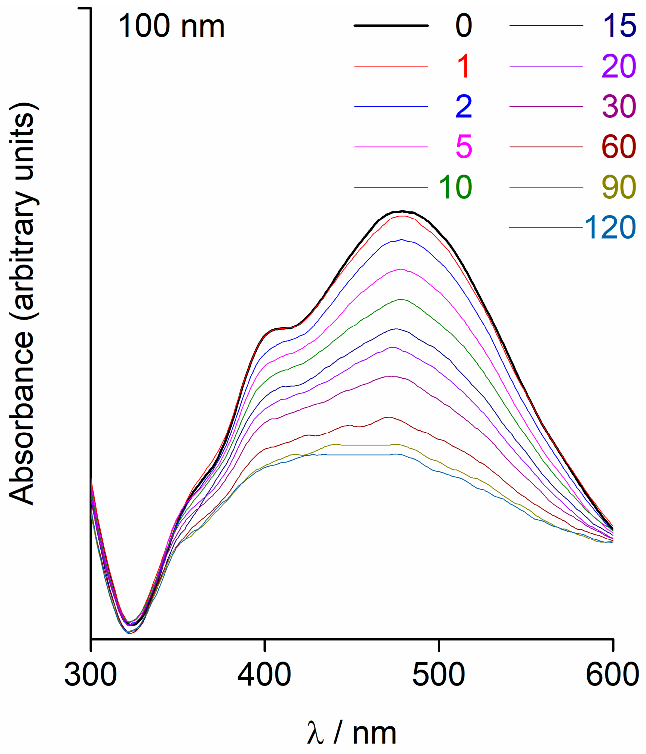

Figure S5. Temporal change in surface plasmon resonance for 100 nm AgNPs (1 mg L^{-1}) in FSW. Legend shows time in minutes after addition of AgNP to FSW.

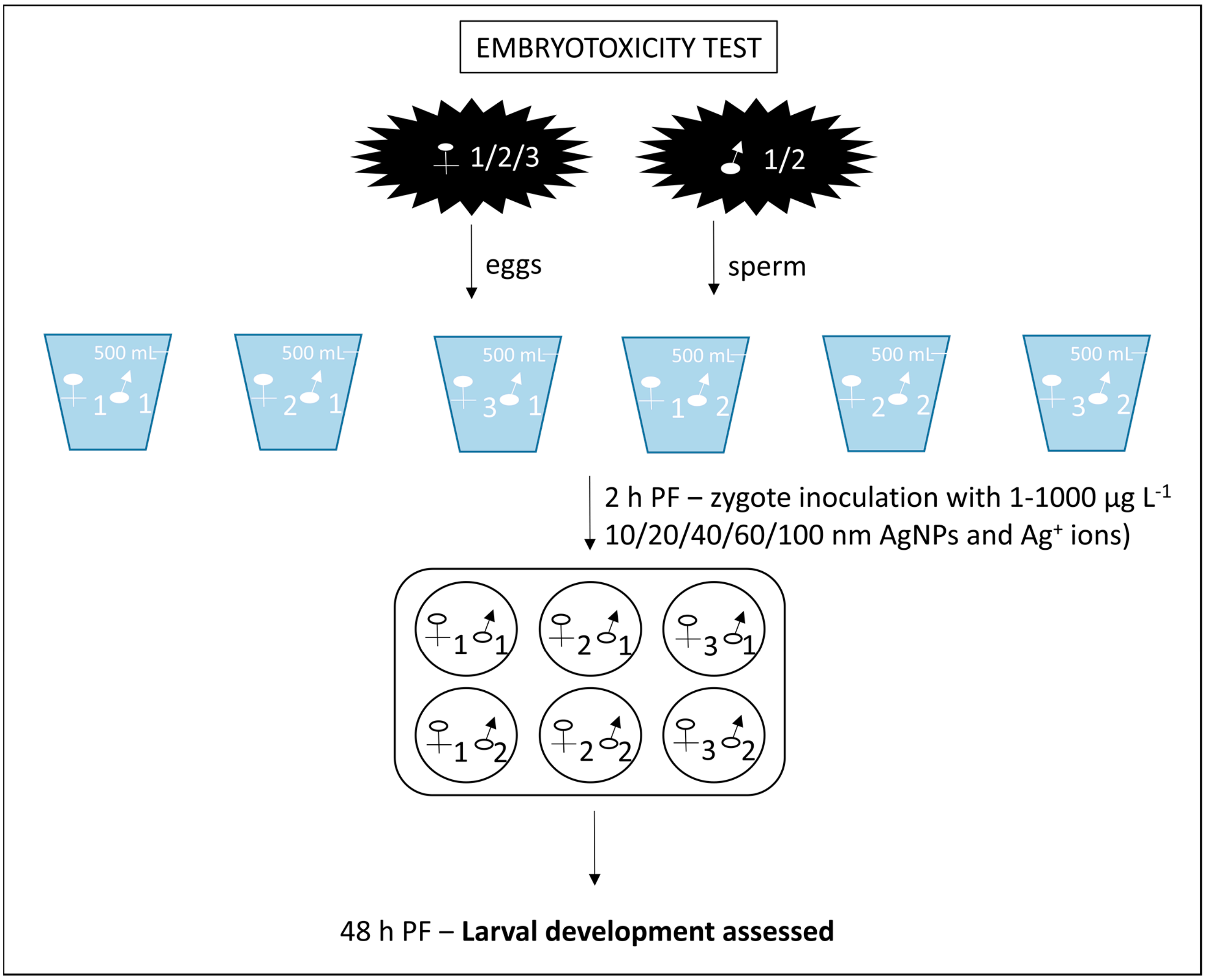

Figure S6. Schematic of the embryotoxicity assay workflow.

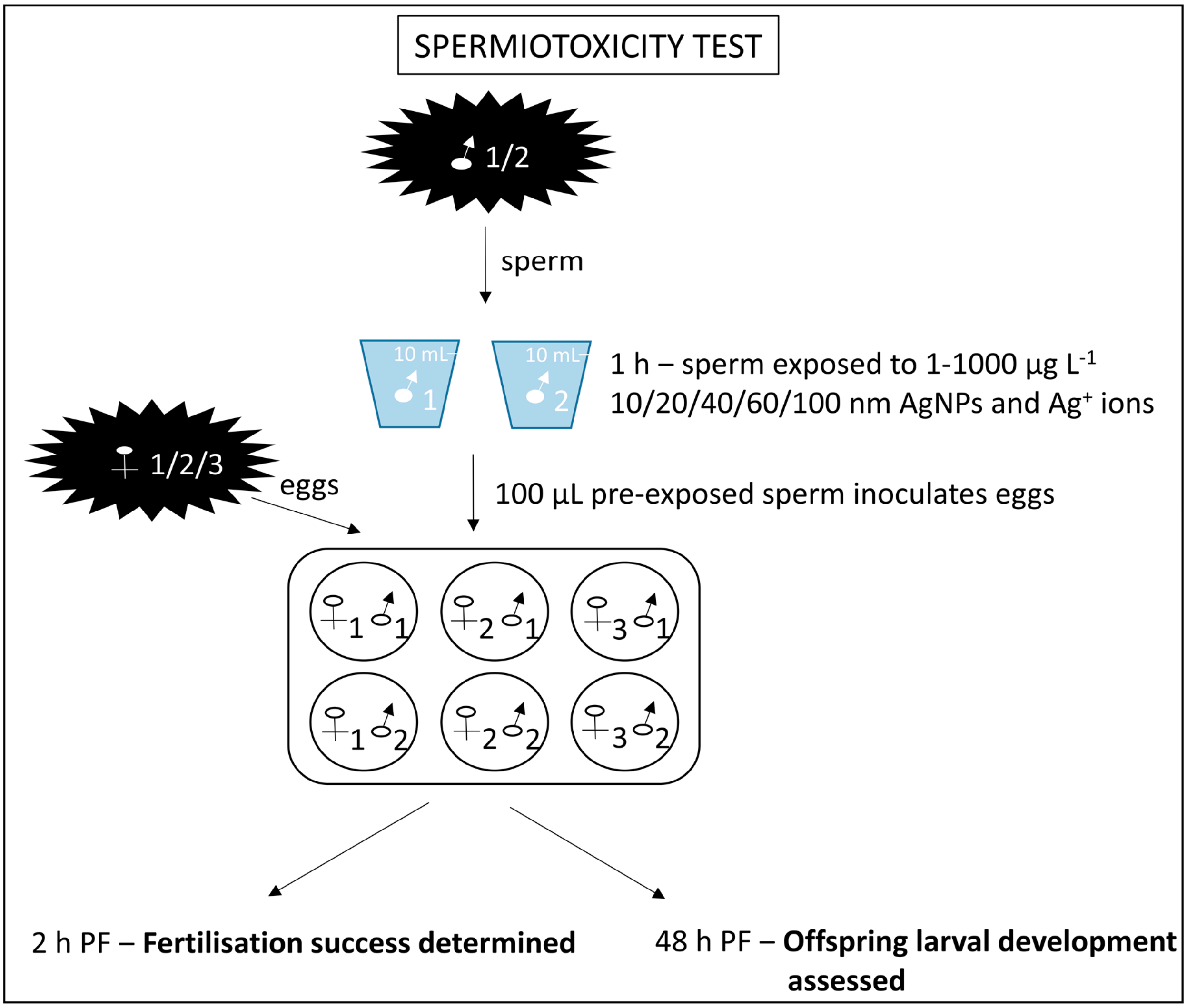

Figure S7. Schematic of the spermiotoxicity assay workflow.
